# SWAT 84: effects of same-day consent vs delayed consent on the recruitment and retention of trial participants—an observational SWAT

**DOI:** 10.1186/s13063-023-07727-w

**Published:** 2023-10-25

**Authors:** M. Elfghi, F. Jordan, S. Sultan, W. Tawfick

**Affiliations:** 1 School of Medicine, University of Galway, University Road, Galway, Ireland; 2 Department of Vascular and Endovascular Surgery, University College Hospital, Galway (UCHG), Newcastle Road, Galway, Ireland

**Keywords:** Adherence, Consent, Delayed, Dropout, PAD, Same-day, SWAT, Withdrawal

## Abstract

**Background and aim:**

The recruitment process in a randomized trial can be challenging. Poor recruitment can have a negative impact on the allocated budget and estimated completion date of the study and may result in an underpowered study. We aimed to perform a Study Within A Trial (SWAT) to evaluate the impact of same-day consent or delayed consent on recruitment and retention in the host trial.

**Methods:**

This SWAT is designed as a prospective cohort design. The host trial was a randomized controlled trial evaluating the effectiveness of an intensive lifestyle modification programme in participants with peripheral arterial disease. Researchers screened the participants for inclusion and exclusion criteria. Informed consents were obtained from the participants who were willing to participate in the study on a standardized consent form. Participants were given the option to consent on the same day or to delay their consent. Following the consent, the participants were allocated to two groups (same-day consent vs. delayed consent) based on pre-determined criteria for SWAT. One hundred sixteen participants were consented to take part in the host trial. Seventy-five participants were randomized to the host trial. The primary outcome was the proportion of participants who withdrew consent at the recruitment phase. Secondary outcomes were reasons for consent withdrawal and dropout, attrition rate, and adherence with the host trial intervention.

**Results:**

There was a significantly lower consent-withdrawal rate in same-day consent (17.4%, *n* = 8/46), compared to the delayed consent group (47.1%, *n* = 33/70), *p* = 0.001. There was a significantly lower dropout rate in participants randomized following same-day consent (10.5%, *n* = 4/38), compared to those randomized after delayed consent (29.7%, *n* = 11/37), *p* = 0.038. Transport was the main reason mentioned for consent withdrawal and dropout. In participants randomized to the host trial intervention arm, there was a significant difference in adherence (percentage of the 12-week programme completed) between same-day consent (96.7% ± 4.9) and delayed consent participants (86.4% ± 11.2), *p* = 0.003, as well as number of weeks completed (mean difference =  − 1.547, 95% confidence intervals (− 2.237 to − 0.85)), *p* = 0.02.

**Conclusion:**

This SWAT found evidence that participants who gave consent on the same day seemed to have better adherence and fewer-withdrawal and dropout rates.

**SWAT registration:**

The SWAT was registered on the Northern Ireland Network for Trials Methodology Research, SWAT 84.

## Background

Randomized controlled trials (RCTs) are widely acknowledged as the design of choice for evaluating the effectiveness of healthcare. Methods to increase recruitment in randomized trials are priorities for methodological research [1]. The success of RCTs depends on the recruitment and retention of trial participants [2]. However, the recruitment process in RCTs can be challenging for the researcher. At least 50% of trials fail to recruit the required sample size, leading to an underpowered study and minimizing the internal and external validity of trial [3, 4].

Informed consent is an important part of ethical clinical research. The consenting process is both legal and ethical in nature requiring a lot of consideration during the trial design phase. According to the International Conference on Harmonization Guideline for Good Clinical Practice (ICH GCP) [5], trialists should ensure that participants being recruited to join a study should be given adequate and reasonable time to think, prior to consenting to join the study. There is no clarification as to what is considered to be a reasonable time. The timing of the consenting process and its impact on trial adherence and retention is poorly understood.

Low patient retention rates and the consequent missing data during trial follow-up can compromise the internal and external validity of the trial and introduce attrition bias, leading to inadequate sample sizes and reduced statistical power, which can affect the reliability and validity of outcomes [6, 7]. If attrition is less than 5%, it may not result in a concerning bias, and if attrition is between 5 and 20%, it may cause a minor bias, but if attrition is greater than 20%, it can risk the validity of the trial [8]. Poor recruitment and loss of participants can result in increasing the budget and time and may result in an underpowered study that will not adequately answer the original research question [2].

Several studies evaluated the consent process when recruiting participants for a trial. Grady et al. claimed that concise consent had no significant effect on improving the comprehension or satisfaction of the participants [9]. Other studies have found that it is critical for researchers during the consent process to avoid imposing undue influence on the participants such as restricting time limits to make a decision or sign consent forms before they have had a chance to fully understand the study’s purpose and risks that might impose pressure on the participants to join the study and undermine the voluntariness of their decision to participate in the study [10, 11].

More studies are required to identify strategies to improve recruitment and the consent process within randomized trials. While these studies may only have modest effectiveness, together they could have a crucial impact on the costs or duration of a study. According to a survey performed in the USA of 1024 clinical research coordinators, the length of the clinical trial was the main factor in poor retention (60%), followed by participants’ perceptions of the infectiveness of investigational interventions (43%) [12]. Research has shown many effective recruitment and retention strategies in clinical trials which include engaging stakeholders, designing a pragmatic and simple protocol, selecting appropriate sites and communicating effectively with potential participants. The use of technology, such as artificial intelligence, can also improve recruitment and retention [7]. Yet, to our knowledge, there is a paucity in the literature regarding the effect of same-day and delayed consent on the recruitment and retention of trial participants.

There is an argument to be made that a participant who consents on the same day could be more determined to join as they already understand the benefits of the study, whereas undecided participants would tend to delay their joining and probably not be fully convinced of the benefits, causing higher attrition rates. There is, however, a counterargument that participants who took longer to give their consent, only gave their consent after full studying of the material and without feeling coerced thus enabling the participant to make an informed decision to participate, making them more determined to continue with the study with less attrition. Thus, there is a need to study whether or not the timing of consent has any impact on retention and participation in a clinical trial.

### Objectives

We aimed to perform a Study Within a Trial (SWAT) to evaluate the impact of same-day consent or delayed consent on recruitment and retention in the host trial.

## Methods

The protocol design for this study within a trial (SWAT) was previously published in Wiley Online Library and registered at the Northern Ireland Network for Trials Methodology Research SWAT Store (SWAT84; 1/SEP/2018). Ethical approval has been obtained from the Merlin Park Hospital, Clinical Research Ethics Committee (approval number: C.A. 1912) [13].

A SWAT is a self-contained research study design that has been embedded into a larger host trial for the purpose of assessing or investigating alternative ways to carry out and improve evidence base concerning trial processes [14].

This SWAT was a part of a study evaluating the effectiveness of an intensive lifestyle modification programme in patients with peripheral arterial disease, from here on referred to as ‘the host trial’. The host trial was a two-armed randomized controlled trial of a risk factor modification intervention programme versus standard healthcare in a tertiary vascular care centre.

The SWAT itself was designed as a prospective cohort design, non-randomized, parallel study to evaluate the effect of same-day consent compared with delayed consent on recruitment and retention of host trial participants.

### Host trial design

The host trial is an RCT evaluating the effectiveness of an intensive lifestyle modification programme in participants with peripheral arterial disease (PAD). Participants who met the eligibility assessment criteria were identified from the outpatient PAD clinic at the University Hospital Galway, Ireland (UHG). Initial screening and randomization were carried out at the outpatient PAD clinic in UHG. The risk factor modification intervention programme was delivered in a nurse-led community-based centre (Croí Heart and Stroke Centre, Galway, Ireland), in the presence of a physiotherapist and nutritionist. The outpatient PAD clinic in UHG managed the control arm. The Department of Vascular and Endovascular Surgery, UHG, and the School of Medicine at the University of Galway served as the co-ordination centre. Patients were directly supervised during the intervention. The 12-week and 1-year follow-up examination and assessment took place at the outpatient PAD clinic in UHG [15].

Eligibility criteria for inclusion and exclusion in the host trial have been mentioned in “The effect of lifestyle and risk factor modification on occlusive peripheral arterial disease outcomes: standard healthcare vs structured programme for a randomised controlled trial” protocol [15]. There were no separate inclusion or exclusion criteria necessary for SWAT.

Researchers screened the patient for inclusion and exclusion criteria. All potential participants with symptomatic PAD (Rutherford category 2 and above) [16] were invited to join the host trial. The host trial is a two-armed randomized controlled trial of a risk factor modification intervention programme versus standard healthcare in a tertiary vascular care centre. Invited patients were provided with a pre-designed information leaflet. This leaflet was fully explained to the patient at the initial assessment. The study researchers have answered any questions about the study prior to participation in the trial regarding the diagnosis, aim and nature of proposed therapy, possible risks and advantages of the treatment, purpose and design of the study trial, techniques used for treatment allocation (i.e. randomization), any further tests or procedures necessary and rights of study participants [17]. Informed consents were obtained from the participants who were willing to participate in the study on a standardized consent form following ICH GCP guidelines [5]. Participants were given the option to consent on the same day or to delay their consent. Following the consent, the participants were allocated to two groups (same-day consent vs. delayed consent) based on what they opted for. Participants were given an appointment for randomization and baseline assessment for the host trial.

After meeting the inclusion criteria, screened patients were randomized to one of two treatment arms. One arm received the 12-week intensive risk factor modification intervention programme. The control arm was provided with standard care in the outpatient PAD clinic. The risk factor modification programme involved a weekly visit for 12 weeks and follow-up appointment for one year, whereas for standard care participants were given advice on the risk factor adjustment and reviewed at 12 weeks and followed up at 12 months.

### SWAT methodology

#### SWAT sample size

There was no formal sample size calculation performed for SWAT, which is in line with SWAT methodology. PROMoting THE USE of SWATs (PROMETHEUS), a national initiative by UK research and innovation acknowledges that sample size can be an issue for SWATs as they are constrained by the host trial sample size, so a separate power calculation is not useful or necessary [18]. Even though individual SWATs might lack the statistical power a meta-analysis of similar SWATs can provide compelling evidence. Thus, the sample size of our SWAT was limited to the number of participants willing to sign the consent [14, 19]. This is an interim analysis of the host trial once we reached 33% of the power of the study. There were no additional inclusion and exclusion criteria for SWAT.

#### SWAT study design

The purpose of the SWAT was to determine the effect of same-day vs delayed consent on recruitment, retention/attrition, and adherence of the participants in the host trial. The study was designed as a prospective cohort study. We followed the Strengthening the Reporting of Observational Studies in Epidemiology (STROBE) statement guidelines for reporting on observational studies [20]*.*

After explaining the host trial and SWAT study to the invited patients with PAD, the patients who agreed to participate in the trial were given the option to sign the informed consent form on the same day or to delay signing their consent prior to alter date. Following the consent, the participants were assigned one of two groups (same-day consent vs. delayed consent) based on the participant’s choice of signing the consent form. After the participants consented to join the host trial, they were then randomized to either the control arm or the intervention arm of the host trial. This randomization within the host trial was independent on the fact that the participants had given a same-day or a delayed consent.

#### Same-day vs delayed consent

##### Same-day consent group

The standard informed consent procedures used in the hospital for clinical trials were followed for SWAT participants prior to participation in the trial. The participants who chose to give consent on the same day following the initial meeting, after the pre-designed information leaflet for the host trial had been fully explained by the investigator were placed in the same-day consent group.

##### Delayed consent group

The standard informed consent procedures used in the hospital for clinical trials were followed for SWAT participants prior to participation in the trial. The participants who chose to give consent on the next day or following the initial meeting were given an unsigned consent form, with an addressed and stamped envelope, after the pre-designed information leaflet for the host trial had been fully explained by the investigator and were placed in a delayed consent group. Participants were allowed time to discuss with family and friends and were advised to ring the investigators with their verbal consent and send back the signed written consent form, only when they feel comfortable joining the study. The investigator called the participant on the third day after the initial meeting (if the returned envelope had not arrived), to ask if they had decided to join or not. Participants were not coerced into consenting to participate at any time.

### Outcomes

#### Primary outcome

The primary outcome measured the effect of same-day consent on the recruitment and retention of participants as compared to the delayed consent. As such, the primary outcome is the difference in the proportion of participants who have withdrawn the consent, if they had initially signed the consent form either on the same day or delayed, and then withdrew that consent prior to the baseline assessment and randomization meeting.

#### Secondary outcomes

The secondary outcomes included:

1. Reasons for withdrawal of consent; this stage can be prior to randomization in the host trial, at the time of randomization or baseline assessment before commencing treatment in either the intervention or comparator arm in the host study.
2. Attrition rate following commencement of host trial; This was calculated as the percentage of randomized participants who did not complete the study and opt out of the host trial prior to the completion of the 12 weeks and did not attend the 12-week assessment.
3. Reasons for drop out from host trial.
4. Adherence rates within the host study trial; this included the percentage of the 12-week programme completed by intervention participants**.** This outcome was only assessed in participants who were randomized to the intervention arm of the host trial.

### Statistical analysis

The baseline characteristics of participants in each SWAT intervention were analysed using descriptive statistics. Numbers and percentages within the same-day and delayed consent study groups were reported for categorical outcomes. All collected data was analysed using IBM Statistical Package for the Social Sciences (SPSS) statistics version 28 software (IBM, Armonk, NY, USA). A two-sided *p* value of < 0.05 was taken to indicate statistical significance. The primary outcome was assessed using a chi-square test to determine the difference of same-day consent versus delayed consent in recruitment and retention rate. The quantitative analysis of secondary outcomes was assessed using descriptive statistics such as the frequency and percentage of participants citing reasons for withdrawal of consent and drop out from the host trial, attrition rate following the commencement of the host trial and compliance with host trial intervention.

## Results

Participants’ recruitment took place between 17/05/2019 and 24/09/2021. A total number of 435 PAD participants were eligible to be included in the host trial. Two hundred fifteen PAD participants agreed to discuss the trial with the investigator and were given the option to either consent of the same day of their visit or they could take time to decide. A total of 116 (54%) participants were interested in taking part in the host trial while 99 (46%) either declined or did not respond to take part in the host trial. Participants who agreed to consent were then assigned to one of the two groups according to pre-defined criteria, i.e. same-day consent group and delayed consent group. Forty-six out of 116 participants agreed to consent and opted to sign the written consent at the setting on the same day and were placed in the same-day consent group, while 70 out of 116 participants agreed to consent and opted to take time and return the consent at a later stage and were placed in the delayed consent group. 65% (46/70) of the participants in the delayed consent group were reminded by the investigator for not returning the consent form, three days following the initial meeting. Thirty-seven out of 70 participants in the delayed consent group returned the signed written consent form. Overall, 41 out of 116 participants from both groups decided to withdraw their consents at the recruitment phase of the host trial prior to randomization in the host RCT. There was a significantly lower consent-withdrawal rate in the same-day consent group (17.4%, *n* = 8/46), compared to the delayed consent group (47.1%, *n* = 33/70) *p* = 0.001.

The reasons mentioned by the participants for withdrawal of consent at the recruitment phase prior to randomization in the host trial included transport (*n* = 15), distance (*n* = 11), lack of family support (*n* = 3), busy schedule (*n* = 5) and loss of interest (*n* = 1) whereas, six participants did not respond when the investigator called them back. These participants were not included in the host trial.

Overall, 75 PAD participants from the time of initial assessment in both the same-day consent group and delayed consent group were randomized to either treatment or control arm in the host trial. The mean age across the host trial population was 64.3 ± 7.2, 56%, of which, 56% were males (*n* = 42). Demographic data of the host trial and the SWAT are shown in Tables 1 and 2.

**Table 1:** Participant population demographics for control vs intervention in the host trial

Demographics	Control (*n* = 36)	Intervention *(n* = 39)
Age (years)	64.53 ± 7.94	64.15 ± 6.55
Gender (male)	19 (52.7%)	23 (58.9%)
Smoking status (smoker)	20 (55.5%)	20 (51.2%)
Hypertension	23 (63.8%)	20 (51.2%)
Diabetes mellitus	27 (75%)	19 (48.7%)
Hyperlipidaemia	28 (77.7%)	23 (63.8%)
CAD	10 (27.7%)	12 (30.7%)
Heart failure	4 (11.1%)	4 (10.2%)
CVA	4 (11.1.0%)	4 (10.2.%)

*CAD* coronary artery disease, *CVA* cerebral vascular attack

**Table 2:** Participant population demographics for SWAT

Demographics	Same-day Consent group (*n* = 38)	Delayed Consent group (*n* = 37)
Age (years)	64.24 ± 8.27	64.41 ± 6.03
Gender (male)	23 (60.5%)	19 (51.3%)
Smoking status (smoker)	20 (52.6%)	20 (54.04%)
Hypertension	18 (47.3%)	25 (67.5%)
Diabetes mellitus	25 (65.7%)	21 (56.7%)
Hyperlipidaemia	28 (73.6%)	23 (62.1%)
CAD	17 (44.7%)	5 (13.5%)
Heart failure	5 (13.1%)	3 (8.1%)
CVA	2 (5.2%)	6 (16.2%)

*CAD* coronary artery disease, *CVA* cerebral vascular attack

**Fig. 1:**
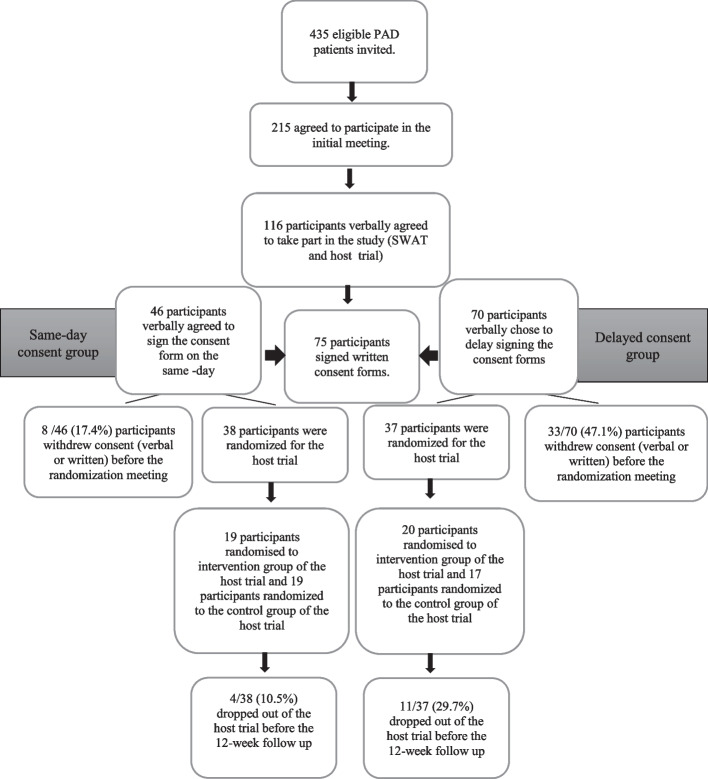
Flow chart for the SWAT study

In the intervention arm, out of 39 participants, 19 were from the same-day consent group and 20 belonged to the delayed consent group. While in the control arm, out of 36 participants, 19 were from the same-day consent group whereas 17 were from the delayed consent group. The SWAT flow diagram is shown in Fig. 1.

After randomization, 15 participants dropped out from the host trial study. Fourteen participants dropped out from the intervention arm of the host trial and only one participant dropped out from the control arm. There was a significantly lower attrition rate in participants randomized following same-day consent (10.5%, *n* = 4/38), compared to those randomized after delayed consent (29.7%, *n* = 11/37), *p* = 0.038. Transport (*n* = 7) and lack of family support (*n* = 2) were the commonest reasons for attrition in the host trial. Other reasons for attrition included distance (*n* = 1), loss of interest (*n* = 1), unsuitable timetable (*n* = 1) and the difficulty level of intervention (*n* = 1), yet two participants did not respond when the investigator called them back.

Out of 39 participants who were randomized in the host trial to the intervention arm, there was a significant difference in adherence (percentage of the 12-week programme completed) between same-day consent participants (96.7% ± 4.9) and delayed-day consent participants (86.4% ± 11.2), *p* = 0.003.

In the same group of participants who were randomized to the host trial intervention arm, there was a significantly higher number of weeks completed by participants following same-day consent when compared to delayed consent (mean difference =  − 1.547, 95% confidence intervals (− 2.237 to − 0.85), *p* = 0.02.

## Discussion

Evidence in the literature has evaluated the effect of the consent process on different aspects of research methodology [10]. The present study aimed to investigate the impact of same-day consent versus delayed consent on the recruitment and retention rates, as well as adherence to intervention in a randomized controlled trial (RCT) among patients with peripheral arterial disease (PAD). The results showed that same-day consent was associated with a significantly lower consent-withdrawal rate and attrition rate, as well as higher adherence to intervention, compared to delayed consent.

One of the key advantages of same-day consent is that it allows potential participants to make an informed decision about their participation in the trial while they are still present in the clinic, which may increase their motivation and engagement [21]. This is particularly important for patients who may face a range of barriers to participation including transportation issues, lack of family support, and competing demands on their time. By providing patients with the opportunity to discuss the trial with the investigator and sign the informed consent form on the same day, this may increase the likelihood of recruitment and retention.

Moreover, same-day consent was also associated with a significantly lower attrition rate in the host trial. Our SWAT found that there was a significantly lower consent-withdrawal rate in same-day consent participants when compared to the delayed consent participants. This might mean that participants who consent on the same day could be more determined to join the study. Yet, participants who decide to delay their consent might not be fully convinced of the benefits, causing withdrawal of their consent. The most common cause for consent withdrawals among the participants was transport.

Attrition is a common problem in research studies and can undermine the validity of the study findings if it is not addressed properly [22]. Even though participants took their time to further think about joining and decided to participate in the trial, their dropout rate was higher. Our finding that same-day consent was associated with lower attrition rates may be due to the fact that participants who were able to provide immediate consent may have been more motivated or committed to the study or may have better understanding about the study and their role in it. Similarly, the most common cause for dropout among the participants was transport.

Furthermore, same-day consent may help to establish a stronger rapport between the patient and the investigator, which may enhance patient engagement and adherence to intervention [21]. This is supported by the higher adherence rate in the same-day consent group in the present study. Participants who required more time to think about signing consent have higher withdrawal consent rate and lower adherence with the intervention when compared to same-day consent. This may be due to the reason that patients in the delayed consent group had more time to reflect on their decision to participate in the trial and may have been more likely to experience doubts or concerns about the intervention. By contrast, patients in the same-day consent group may have felt more committed to the trial and more invested in its outcomes.

Rushing participants to consent could be construed as coercion or undue influence. According to the Food and Drug Administration (FDA) document on consent every effort must be made to avoid coercion or undue influence during the consent process [23]. Participants in this SWAT who gave their consent on the same day seem to have better adherence and less consent withdrawal and dropout rates. This could suggest that even if the participant consents on the same day, it will not necessarily mean that coercion or undue influence was involved, as long as proper due diligence is followed during the consent process, informing the participant in full detail of the pros and cons of taking part in the study. However, we have to take into account that participants not being able to take some time away to consider whether or not they want to take part in a study is still considered an ethical issue with recruitment [10].

Despite these advantages, there are some potential drawbacks to same-day consent that should be considered. For example, patients may feel pressured to participate in the trial if they are asked to sign the consent form immediately, which may compromise their autonomy and informed consent [24]. Moreover, patients may not have sufficient time to review the trial information or discuss it with family members or other healthcare providers, which may lead to misunderstandings or regrets later on [25]. However, the results from our study have proven that these factors have a smaller influence on the attrition rate.

### Limitations

Despite the strengths of SWATs, there are several limitations that need to be acknowledged. The sample size of this study was relatively small, as it was a single-centre study which may have limited the generalizability of the findings to other populations. The major weakness that was associated with this SWAT was that several participants decided not to join the study or left the study for other reasons that were not related to the timing of their consent. We tried to establish this information, yet there was a possibility that participants who withdrew their consent or dropped out from the host trial were not comfortable to convey the real reasons for doing so, and others did not respond back when they were called back by the investigator. Another sample-related limitation is the observational approach of the study. The participants self-selected their choice of consent, thus rather than being the cause of better outcomes it may be the predictor of outcomes. We acknowledge that non-randomization may introduce selection bias. However, we aimed to mimic the real-life consent process as much as possible, without putting the participants under pressure. The rationale behind not randomizing the timing of consent was to avoid coercing participants into having to give an immediate consent if they were randomized to that arm.

We acknowledge that this SWAT is an observational study and may be limited by confounding factors. Nevertheless, the baseline characteristics of the participants included in the SWAT show that there was no difference in the mean age, gender distribution or the risk factor burden between both groups. Furthermore, all participants have the same pathology, so there is no difference in the urgency of the treatment. However, not all confounding factors could be accounted for, as limitations secondary to decisiveness, health literacy, and trust in medical research were not assessed at baseline. We acknowledge that these and similar confounding factors may have affected the outcomes. Future studies should be developed to address these factors along with consent choice.

Admittedly, the outcomes of such an observational approach may not necessarily be generalizable to clinical trials where participants do not have a choice to select the consent method. However, they still provide meaningful, real-life information regarding participants’ behaviour.

## Conclusion

Overall, the findings of the present study suggest that same-day consent may be a useful strategy for improving recruitment, retention, and adherence rates in RCTs among patients. (Or it can be a predictor of a higher retention rate and lower attrition rate) The participants who consented on the same day showed significantly lower consent-withdrawal and dropout rates and a better adherence.

## SWAT registration

The SWAT was registered on the Northern Ireland Network for Trials Methodology Research [26]. The protocol of this *SWAT* was published [13]. The host trial was registered (11/07/2017) on the European Clinical Trials Database (EudraCT number 2017–002964-41) and ClinicalTrials.gov (NCT03935776).

## Data Availability

The final datasets underlying publications, resulting from this SWAT, will be shared as an anonymous copy upon reasonable and approved request. A request may be made through email to the Principal Investigator and can only be made upon meeting the terms and conditions for the ethics approval of this trial.

## Abbreviations

CAD: Coronary artery disease
CVD: Cerebral vascular attack
FDA: Food and Drug Administration
ICH GCP: International Conference on Harmonization Guideline for Good Clinical Practice
PAD: Peripheral arterial disease
RCT: Randomized controlled trial
SWAT: Study within a trial
UHG: University Hospital Galway, Ireland
ICH: International Conference on Harmonisation
